# Modifying the potato tuber storage protein patatin targeting improved thermal stability

**DOI:** 10.1007/s00425-025-04766-2

**Published:** 2025-07-11

**Authors:** Martin Friberg, Shrikant Sharma, Folke Sitbon, Mariette Andersson, Per Hofvander

**Affiliations:** 1 Department of Plant Breeding, Swedish University of Agriculture, Alnarp, Sweden; 2 Department of Plant Biology, Swedish University of Agriculture, Uppsala, Sweden

**Keywords:** Differential scanning fluorimetry, Gene editing, Patatin, Potato genetics, Protein engineering, Storage protein

## Abstract

**Main conclusion:**

Gene editing of the patatin gene cluster using a single-guide RNA sequence consistently modifies over 10% of the targeted genes in modified individuals.

**Abstract:**

Patatins have gained recent attention, as a group of highly nutritious proteins with excellent functional properties. Some techniques have been suggested for industrial-scale patatin purification, mostly as a by-product from potato starch processing. The purification process has proved to be a challenge due to the low thermostability of patatins, especially under acidic conditions. One strategy to make patatin more accessible for extraction would be to stabilize the protein structure through the introduction of point mutations. Here, we show that the tuber expression of patatin genes is dominated by a few genes from the extended gene family, most of which were predicted to be catalytically inactive. We have further evaluated the suitability of the patatin gene cluster as a target for clustered regularly interspaced repeat (CRISPR)/Cas9-based mutagenesis. In the mutation study, we show that targeting using a single single-stranded guide RNA (sgRNA) can lead to mutations in over 10% of all alleles. Finally, four patatin variants with amino acid substitutions were designed based on in silico analysis of patatin protein structure. These modified patatins were then heterologously expressed in bacteria and evaluated for increased thermostability. While none of the mutant proteins performed better than a wild-type variant, with regard to their thermal properties, one candidate proved to be less sensitive to shifting pH, making it an interesting candidate for further optimizations.

**Supplementary Information:**

The online version contains supplementary material available at 10.1007/s00425-025-04766-2.

## Introduction

In recent years, the popularity of plant-based proteins in food products has increased significantly. This in turn has led to an expansion in applied research. While ample focus has been devoted to processing methods for development of new meat substitutes (Andreani et al. 2023), little attention has been given to novel protein sources. A crop species that is rarely mentioned in the plant protein context is potato (*Solanum tuberosum*). Protein constitutes only about 2% of the fresh weight in potato tubers (Luo et al. 2024), which is low compared to the seeds of classical protein crops. However, potato is a high yielding crop with an average yield of over 20 tons per hectare globally (Food and Agriculture Organization of the United Nations 2024), and over 37 tons per hectare in Europe (Eurostat 2024). Potato, therefore, has a potential protein yield of 400–700 kg protein per hectare, which can be compared with the 700–1500 kg per hectare seen in cultivation of soybean in Europe (Karges et al. 2022).

In addition to being grown for various food uses, potatoes are used for starch extraction. Starch potatoes are mainly grown in Northern Europe and some additional regions in the world. In Europe, 15% of the total starch production comes from potatoes (Starch Europe, 2022, starch.eu). During starch processing, the tuber proteins mainly end up in the potato fruit juice (PFJ), a side-stream also high in glycoalkaloids that are toxic compounds. This side-stream is commonly treated with a combination of heat and acidic conditions, to coagulate and precipitate the proteins, which are subsequently dried to a powder. This protein powder is used as animal feed as high content of glycoalkaloids makes it unsuitable for human consumption (Løkra and Strætkvern 2009).

The potato tuber protein extract mainly consists of two types of proteins, i.e., protease inhibitors, which make up about 30% of total native protein (Melville and Ryan 1972), and patatins that make up between 25 and 45% (Racusen and Foote 1980; Barta et al. 2012). The patatins are a group of glycoproteins, which predominantly serve as storage proteins in potato tubers. They have a pI of 4.8–5.2 and a molecular weight of 40–42 kDa but are natively present as 80 kDa dimers. In addition to their role as storage proteins, the patatins exhibit a broad spectrum of lipid-acyl hydrolase activity on multiple substrates, including phospholipids (Galliard 1971; Anderson et al. 2002), as well as mono- and di-acylated glycerols (Hirschberg et al. 2001). Hydrolysis is mediated by a dyad active site consisting of a Ser-Asp instead of the more common Ser-His-Asp/Glu triad. In addition, the structure of patatins lacks an amino acid sequence, which forms a lid that usually covers the active site of similar lipases, causing hydrophobic patches of the active site to be exposed (Rydel et al. 2003). This structure and activity has led to discussions about a possible function of patatins in plant defense, either through signal transduction (Senda et al. 1996), or more directly, as patatin has been shown to inhibit the growth of some larvae (Strickland et al. 1995).

On the genomic level, patatins are commonly divided into two classes depending on the absence or presence of a 22 bp sequence located up-stream of the coding sequence. Class-I patatins lack this sequence and are highly expressed in tubers making it the more important group from an industrial perspective, while class-II patatins carry the up-streams sequence, and are expressed at a much lower level and throughout the entire plant (Pikaard et al. 1987; Prat et al. 1990). The class-I patatin genes are present in a highly repetitive gene cluster on chromosome 8, thought to house 21 copies (Yang et al. 2023). However, there is no certainty as to how many of these copies are active, since only seven distinct patatin sequences were found in a cDNA library prepared from cv. Kuras (Bauw et al. 2006). Furthermore, the number and types of patatins expressed has been reported to vary between cultivars (Bauw et al. 2006; Barta et al. 2012) and across developmental stages (Stupar et al. 2006), further adding to the complexity of forming a unifying view on the number and expression of patatin genes in potato.

What makes the patatins attractive for the food industry is their superior amino acid composition in relation to other plant proteins (Van Gelder and Vonk 1980), their high digestibility, and their excellent foaming properties (Ralet and Guéguen 2001; Løkra et al. 2008). All three are properties that make patatins stand out among plant proteins. Thus, it is not surprising that a method based on expanded bed chromatography has been developed for industrial-scale purification of patatins, which is currently being used commercially (Giuseppin et al. 2008). It has also been suggested that ultrafiltration could be used to purify patatin, although membrane fouling (Eriksson and Sivik 1976) and high levels of anti-nutritional compounds (Wojnowska et al. 2006) have limited the use of this technique industrially (Løkra and Strætkvern 2009).

Since the patatin fold structure is highly affected by both pH and temperature, with partial unfolding occurring already at 28 °C (Pots et al. 1998a, b), it would be interesting to study stabilized variants of patatin to see how processability is affected. While thermostability is a complex topic that is not well understood, there are some guidelines for what influences the stability of a protein (Jaenicke and Böhm 1998; Sterner and Liebl 2001). Generally, proteins are structured around a hydrophobic core, with hydrophilic residues exposed to the surrounding solvent. The protein is further stabilized by hydrogen bonds, between amino acid neighbors that are structurally close forming secondary structures such as α-helices, or between residues further apart, as seen in β-sheets (Tompa et al. 2016). There are a number of other interactions that can stabilize a protein, such as cysteines forming di-sulfide bridges, and aromatic amino acids forming pi-stacks, although these are less universal. On the other hand, exposed hydrophobic residues or hydrophilic residues present in hydrophobic pockets introduce tensions to the structure which can lead to reduced stability (Jaenicke and Böhm 1998). A number of post-translational modifications can also affect stability, such as methylation, or in the case of patatin, glycosylation (Shental-Bechor and Levy 2008; Lee et al. 2023).

While many proteins have been engineered for thermostability, such as polymerases for PCR, these are mostly heterologously expressed (Turner et al. 2007). In theory, the same type of modifications should be possible to introduce in situ in plants to improve the stability of plant protein for extraction and purification. Several techniques for gene editing are available, the most prominent in recent years being clustered regularly interspaced repeats (CRISPR) and CRISPR-associated protein (Cas)-based methods. The Cas-proteins are a family of RNA-guided endonucleases, where the most widely used is Cas9 (Chen et al. 2019). While Cas9 has mostly been used to generate knock-out mutations, methods that allow for more precise tailoring of gene edits are being developed. Two prominent examples are base editors (Zong et al. 2017) and prime editors (Anzalone et al. 2019), both of which have been adapted for use in plants although with varying efficiency (Chen et al. 2019; Vats et al. 2024).

In this study, we explored the prospects of modifying a major part of accumulated potato tuber patatin for improved thermostability. Due to the complexity of the patatin gene cluster, several steps of analysis were applied; RNA sequencing data from cv Desirée and cv Kuras tubers were analyzed to determine expression levels of individual patatin genes. A genome editing study utilizing Cas9 was performed to verify the accessibility of patatin genes as targets for more precise gene editing techniques, as well as to assess what fraction of the total number of expressed gene copies could be targeted simultaneously. We performed theoretical modeling, in the form of constraint network analysis, to identify promising areas to introduce amino acid changes and designed four patatin variants to test for increased stability. These variants were heterologously expressed and tested for stability using differential scanning fluorimetry to be used as guiding information in preparation for designing precise gene edits.

## Materials and methods

### RNA extraction, sequencing, and transcript abundance estimation

Two potato cultivars, cv. Desirée and Kuras, were cultivated under controlled greenhouse conditions (16h day, 18°C/15°C day/night temperature, 50% relative humidity, and supplementary light intensity up to approximately 200 µmol m^−2^s^−1^). Tubers were harvested after 4 months and flash frozen in liquid nitrogen before being stored at −80 °C. Total RNA was extracted in triplicates from each cultivar using PureLink™ Plant RNA Reagent (Thermo Fisher Scientific, Waltham, MA, USA) followed by DNase treatment using TURBO DNA-free kit (Thermo Fisher Scientific) as per the manufacturer’s instructions. The concentration and integrity of extracted RNA samples were determined using an Agilent RNA 6000 Nano Kit on Agilent 2100 Bioanalyzer (Agilent, Santa Clara, CA, USA). RNA library construction and sequencing was performed by Novogene (Cambridge, UK). Briefly, mRNA was purified from total RNA using poly-T oligo-attached magnetic beads. First strand cDNA was synthesized using random hexamer primers after fragmentation and followed by second-strand cDNA synthesis. The cDNA samples were end-repaired, phosphorylated, and polyadenylated followed by ligation of adaptors. The ligated fragments were selectively enriched with PCR and pair-end mRNA reads (PE-150) were generated using Illumina NovaSeq6000 S4 sequencing platform. Raw reads were analyzed for read quality with FastQC v0.12.1 (https://github.com/s-andrews/FastQC) and filtered using fastp v0.23.4 (Chen et al. 2018; Chen 2023). The filtered reads were aligned to DM v8.1 potato genome assembly (Yang et al. 2023) using splice aligner STAR v2.7.10b (Dobin and Gingeras 2015) with –twopassMode Basic –outSAMtype BAM SortedByCoordinate, keeping other parameters as default. Transcript abundance was estimated with Salmon v1.10.2 (Patro et al. 2017) in alignment-based mode and reported as TPM values.

### Plant materials and growth conditions

In vitro-cuttings of cultivars, Desirée, and Kuras were kept on a solidified Murashige Skoog medium supplemented with 3% sucrose, in a controlled growth chamber equipped with fluorescent light tubes giving a photon flux of 100 µmol m^−2^ s^−1^, and with a 16 h light/8 h dark photoperiod (24°C).

Plantlets for protoplast isolation were used within 6 weeks of planting. Cuttings were refreshed by removing the top 1–2 cm of the plant and transplanting it in fresh media.

### Protoplast isolation, transfection, and shoot regeneration

Protoplasts isolation, transfection, and regeneration were performed following Nicolia et al. (2021), using leaves from 5–6-week-old Desirée plantlets as starting material. The RNP complex was prepared from 5 µg TrueCut (Thermo Fisher Scientific) and 0.1 nmol synthesized gRNA (GTAATTGGAGGAACAAGTAC, Synthego, Redwood city, CA, USA) that were mixed to a total volume of 2 µL and incubated for 15 min at room temperature (about 20 °C). Transfection was carried out on batches of 100 000 protoplasts, by mixing protoplasts with the RNP complex and 40% PEG4000 for 30 min.

### Preliminary screening of potato shoots by high-resolution fragment length analysis (HRFA)

Leaf pieces of regenerated shoots were sampled into 96-well plates, pre-loaded with glass beads before being freeze-dried for 48h. Samples were then immediately pulverized by shaking at 15 Hz for 30 s, before being stored at −20°C.

Genomic DNA was extracted from pulverized samples using a high-throughput kit from OMEGA Biotech (Norcross, GA, USA). A region flanking the target site of the gene was amplified from the genomic DNA by PCR, using PhireII, a fluorescence tagged forward primer (HEX-GGACAATAATAAAGATGCAAG ACTTG) and unlabelled reverse primer (AGCTGCAAAGGGTCGATT). Cycling conditions were 98 °C 1 min, 31 cycles of 98 °C 5 s, 63 °C 5 s, 72 °C 10 s, and a final elongation step of 72 °C 1 min.

PCR products were diluted 150 times before 0.5 µL was mixed with 0.5 µL size marker (GeneScan LIZ 600, Thermo Fisher Scientific) and 9 µL Hi-Di Formamide (Thermo Fisher Scientific). Samples were denatured at 95 °C for 3 min, and were then cooled on ice before analysis was run on a 3500 Genetic analyser (Thermo Fisher Scientific) following the manufacturer’s instructions. The results were visualized using GeneMarker v3.0.1 (Softgenetics, State College, PA, USA).

### Genotyping of mutant lines

A selection of events displaying a differential HRFA chromatogram in comparison with wild-type control were further characterized by sequencing. Fragments were amplified, using 5 µL of the genomic DNA prepared for HRFA as template, and using indexing primers ordered from Eurofins (Eurofins Scientific, Luxembourg, Luxembourg) with the same forward and reverse sequences as used for HRFA. Reactions were run using Phusion High-fidelity DNA polymerase in HF-buffer, with the following cycling conditions; 98 °C 30 s, 35 cycles of 98 °C 10 s, 61 °C 20 s, 72 °C 20s, and a final elongation step of 72 °C 5 min.

Indexed fragments were pooled and sequenced by Eurofins, using NovaSeq (Illumina, San Diego, CA, USA). Bar-coded paired-read fastq.gz files were imported as two files to Geneious Prime v. 2024.0.2 (Biomatters L2, Auckland, New Zealand). Individual paired-read sets representing the different samples were separated out from the main files using the “Separate reads by barcode” function. The different samples were then analyzed using CRISPResso 2.0 (Clement et al. 2019) to determine mutation frequency and sequence distribution among reads.

### Protein modeling

The published structure of pat17 from *Solanum cardiophyllum* (1OXW) (Rydel et al. 2003) was retrieved from the protein databank (PDB, www.rcbs.org). Primary analysis was conducted using the web-tool CNAnalysis (Radestock and Gohlke 2008; Krüger et al. 2013), using the PDB number as input. From the output, predicted unfolding nuclei were visualized using PyMol (Schrödinger 2015), and colored using the Bang Wong palette (Wong 2011). The *Solanum tuberosum* patatin structure was predicted using Phyre2 web service (Kelley et al. 2015), and the sequence of patatin-2-kuras 2 (Bauw et al. 2006).

### Heterologously produced patatin variants

Recombinant patatin proteins were obtained from GenScript (Piscataway, NJ, USA) using their *E. coli* expression service. The proteins were retrieved as inclusion bodies and were refolded by GenScript, using the method established by Hirschberg et al. (2001), substituting beta-mercaptoethanol for DTT. Five different variants were obtained for this study; Pat_wt (no mutation), Pat_M1(T78C, D215C), Pat_M2(S154V, I164L, N292T, A293D, N314D, S355E), Pat_M3(T78C, S153C, D215C, V221C, D223C, T299C), Pat_M4(M192F, L281F, L285F).

### Differential scanning fluorimetry

Differential scanning fluorimetry was performed using a CFX96 RT-qPCR (Bio-Rad, Hercules, CA, USA) following the manufacturer’s instructions for Thermal Shift Assay. SYPRO Orange (Merck, Darmstadt, Germany) was used as stain and lysozyme (Thermo Fisher Scientific) as reference. Four replicates were prepared for each sample, and unfolding was studied from 15 °C to 95 °C, in Tris-buffer (50 mM Tris, 50mM NaCl) at three different pH, 3.5, 5, and 6.5. Protein samples were sonicated for 10 min in an Ultrasonic bath (Sonorex Digitec, Bandelin Electronic, Berlin, Germany) and were centrifuged for 5 min at 10 000 *g* before being used for differential scanning fluorimetry. The relative fluorescence units (RFU) were exported from the Bio-Rad software and the mean of all replicates were visualized using gnuplot 5.4. The melt temperature was calculated using TSA_CRAFT (Lee et al. 2019).

## Results

### Tuber expression of patatin genes in cultivars Desírèe and Kuras

It is largely unknown to what extent the patatin gene cluster on chromosome 8 varies in structure and number of gene copies among potato cultivars and wild relatives, and to what extent the individual genes are expressed. Two common cultivars, Desirée, a table potato, and Kuras, a starch potato, were investigated for gene presence and expression level of individual genes using RNAseq. The RNAseq data from Desirée and Kuras tubers were mapped onto the DM v8.1 assembly and raw counts were converted into TPM (transcript per million) values resulting in expression levels of 19 out of the 21 putative patatin genes present in DM 8.1 (Yang et al. 2023). The comparison of expression data from mapping on these 19 patatin genes indicates cultivar-specific expression of certain genes (Table 1). Four genes differ significantly in expression between the two cultivars, Pat-9, Pat-14, Pat-2, and Pat-16. The first two being more highly expressed in Desirée and the latter two more highly expressed in Kuras. Several genes are hard to assess due to a high variation among Desirée replicates (Supplementary Information SI, Table S1), thus mappings to Pat-1, Pat-6, Pat-12, and Pat-15 warrant further investigation. Expression of Pat-3, Pat-5, Pat-8, Pat-10, and Pat-17 was not detected in any of the cultivars. Additionally, Pat-4, Pat-7, Pat-13 and Pat-11, Pat-18, Pat-19 were marginally expressed in Desirée and Kuras tuber samples, respectively.

**Table 1:** TPM values (average value from 3 biological replicates, ± SD) for Desirée and Kuras read mapping on DM8.1, as well as *P* value for t test comparing the two cultivars. The comments regard the properties of the protein expressed by the gene in question. TPM values of individual biological replicates can be found in Supplementary Information SI, Table S1

Gene		Desirée	Kuras	Cultivar difference	Predicted protein properties
DM8C08G01480	Pat-1	2159.44 (± 1198.46)	657.67 (± 116.73)	0.0969	Full length
DM8C08G01490	Pat-2	120.79 (± 44.39)	1078.43 (± 132.43)	0.0003	Incomplete duplication of aa 69–110
DM8C08G01500	Pat-3	0.00	0.00		Truncated
DM8C08G01510	Pat-4	3.92 (± 2.29)	0.91		Truncated
DM8C08G01530	Pat-5	0.3 (± 0.27)	0.08		Truncated
DM8C08G01540	Pat-6	4159.96 (± 2458.92)	433.79 (± 52.01)	0.0585	Inactive (S77G)
DM8C08G01550	Pat-7	1.58 (± 1.65)	0.00		Truncated
DM8C08G01560	Pat-8	0.00	0.00		Truncated
DM8C08G01570	Pat-9	404.78 (± 175.64)	6.58 (± 1.24)	0.0172	Truncated
DM8C08G01580	Pat-10	0.17 (± 0.24)	0.00		Truncated
DM8C08G01590	Pat-11	0.53 (± 0.17)	15.43 (± 3.88)		Truncated
DM8C08G01600	Pat-12	880.4 (± 533.93)	63.57 (± 3.47)	0.0570	Inactive (S77G)
DM8C08G01620	Pat-13	16.64 (± 10.30)	0.00		Truncated
DM8C08G01630	Pat-14	1757.38 (± 886.12)	126.62 (± 14.58)	0.0333	Truncated, Inactive (S77G)
DM8C08G01640	Pat-15	769.71 (± 491.23)	34.18 (± 5.98)	0.0605	Inactive (S77G)
DM8C08G01650	Pat-16	57.44 (± 20.97)	901.77 (± 113.33)	0.0002	Large substitution aa 173–221
DM8C08G01660	Pat-17	0.00	0.11 (± 0.16)		Truncated
DM8C08G01670	Pat-18	0.02 (± 0.01)	2.28 (± 0.17)		Insertion (possible 5’-duplication)
DM8C08G01690	Pat-19	0.00	3.38 (± 0.93)		Full length

Previously reported patatin genes from various potato cultivars were retrieved from NCBI (https://ncbi.nml.nih.gov), and the sequences were translated. A multiple sequence alignment was performed using the predicted protein sequences, as well as the amino acid sequences corresponding to the 19 patatin genes reported in DMv8.1. This revealed further sequence variation between patatin genes (Supplementary Information SII). Pat-3, Pat-5, Pat-7, Pat-8, Pat-9, Pat-10, Pat-11, and Pat-17 appear to be truncated and too short to be catalytically active. Pat-4 and Pat-13 on the other hand are full-length sequences, but contain multiple pre-mature stop codons, which is expected to result in severely truncated proteins. Pat-6, Pat-12, Pat-14, and Pat-15 were found to have a previously reported ‘S77G’ mutation in the active site, rendering the protein inactive (Welinder and Jorgensen 2009). Finally, the characteristic N-terminal signal peptide is missing from translations of Pat-2 and Pat-16, the latter of which also contains a large substitution surrounding the catalytically active D215.

### Gene editing of patatin genes in cv Desirée

**Fig. 1:**
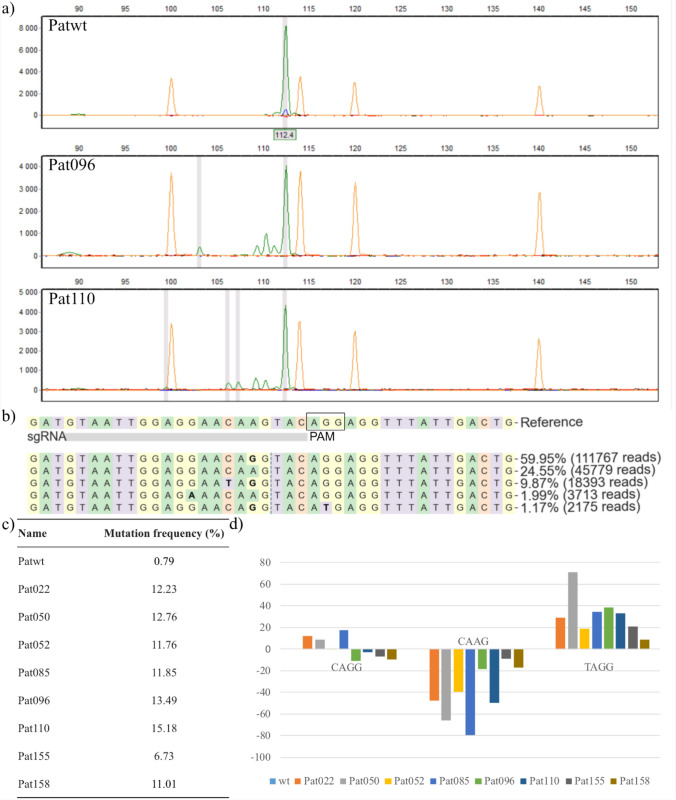
Analysis of patatin gene edited lines generated by RNP transfection. **a** Analysis by high-resolution fragment analysis (HRFA), exemplified by two mutated events (Pat096 and Pat110), as well as the Desirée wt (Patwt). Green peaks represent PCR fragments. Orange peaks are a size ladder. **b** Distributon of wt alleles in Patwt as observed by amplicon sequencing, reference is the sgRNA used to generate mutations, the dotted line indicates the predicted cut site, and missmatches with the sgRNA are highlighted in bold. The PAM-site has been marked with a square. **c** Mutation frequency in sequenced events as estimated by CRISPresso2. **d** Frequency changes of alleles, defined by 4nt sequence up-stream of predicted cut site, in mutant lines as compared to wt

To evaluate the suitability of patatin genes for mutational analysis in planta, a sgRNA was designed to target a conserved area up-stream of the encoded catalytic residue S77. Regenerated events with induced indels in patatin genes were obtained using CRISPR/Cas9 and RNP transfection of protoplasts. After regeneration, shoots were sampled and analyzed using HRFA to identify events with mutations and to determine the size of the induced indels. Several events showed different fragment size-patterns compared to the control, indicating that editing of patatin genes was successful. However, chromatograms of all events analyzed were dominated by a wild-type peak, most likely representing unedited alleles (Fig. 1a). To verify the observed putative edits, and to reveal the relation between edited and the large fraction of unedited alleles, eight events and one wild-type (wt) sample (Patwt) were analyzed using amplicon sequencing. As Fig. 1b shows, a large fraction of the amplified alleles did not perfectly match our targeting sgRNA. Seven of the eight events showed editing in 11–15% of sequenced alleles (Fig. 1c), while one event showed a lower editing efficiency of 6.7%. The allele bias of our designed sgRNA was next investigated by comparing the frequency of wt alleles in the mutated lines, to the frequency in Patwt (Fig. 1d). This showed a decreased presence of sequences that perfectly match the sgRNA, while the frequency of alleles carrying a single mismatch to the sgRNA were largely the same in mutated events as for Patwt. Interestingly, the allele carrying two mismatches compared to the targeting sgRNA increased in frequency among mutated events compared to Patwt (Fig. 1d).

### Patatin protein modeling and design of amino acid exchanges

**Fig. 2:**
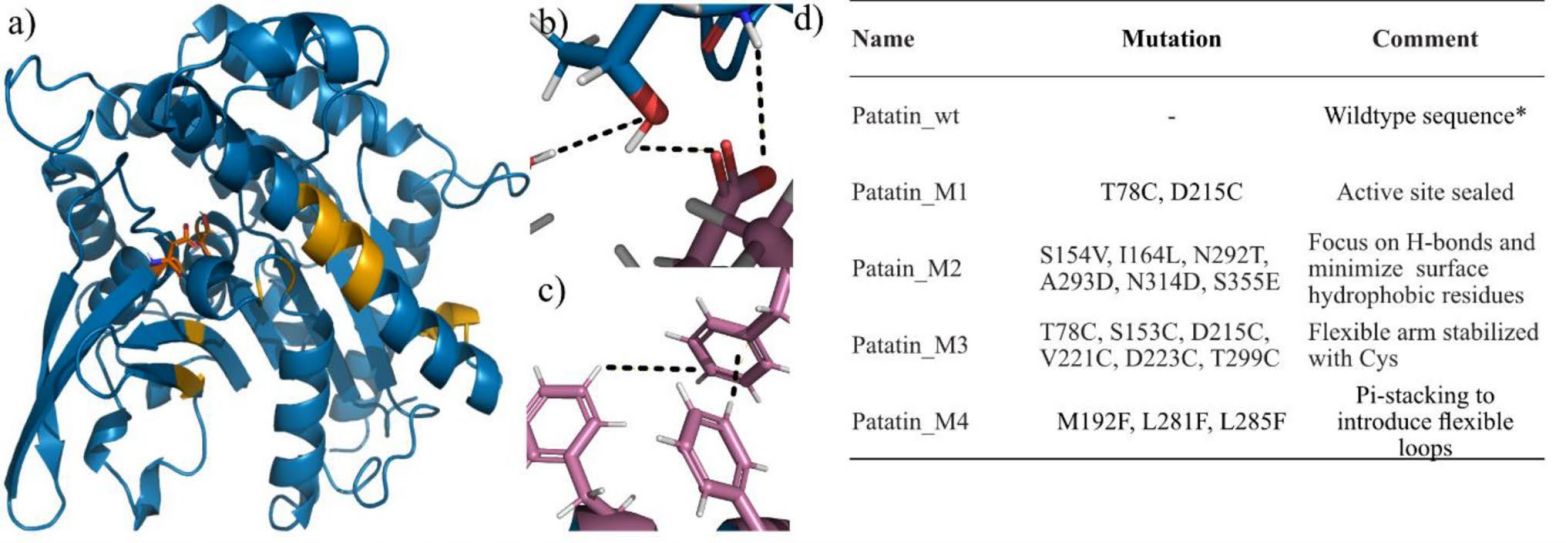
**a** Crystal structure 1oxw with unfolding nuclei predicted by CNA marked in yellow and catalytic residues (S77, D215) marked in orange. **b** Patatin_M2, D293 marked in pink. Dotted lines indicate potential H-bond interactions. **c** Several phenyls are added to a cluster of flexible loops. Dotted lines indicate potential aromatic interactions. **d** List of mutants designed for this study together with some design comments

Since there is no published structure available for patatin from potato, the closely related pat17 (1oxw) from *Solanum cardiophyllum* was used as a model. A primary analysis was performed using constrained network analysis (CNA), utilizing the CNAnalysis web service. The identified unfolding nuclei are highlighted in Fig. 2a. To ensure that there were no major differences predicted in stability between pat17 and patatin in potato, patatin-2-kuras 2 (Bauw et al. 2006) was modeled using the Phyre 2 web server, and this structure was named “Patatin_wt”. The modeled structure was overlaid on top of 1oxw, and the structures and sequences were compared, showing no differences in the unfolding nuclei identified by CNA.

With Patatin_wt as guide, four protein variants were designed with the intent to stabilize different regions of the protein. Some restrictions were used, first to avoid interference with the glycosylation sites since these contribute positively to stability, and second to avoid replacing essential amino acids to preserve the nutritional qualities of the protein. The active site of patatin has previously been identified as a potentially destabilizing region, due to its exposed hydrophobic patches. The two catalytically active residues S77 and D215 were therefore both changed to cysteine residues in an attempt to introduce a di-sulfide bond to close off the active site and to increase rigidity of the core. For the second mutant, the focus was shifted to minimize electrostatic tension in the structure by replacing hydrophobic surface residues with polar alternatives. To ensure that the introduced residues contributed to stability, polar residues were selected on the criterium that they could reach within hydrogen-bonding distance (~ 2 Å) with minimal clashing, an example can be seen in Fig. 2b. The third mutant was a more extensive attempt at stabilizing by di-sulfide bonds with two more potential bonds targeting the beta-sheets to the lower right of Fig. 2a. Finally, for the fourth mutant, three residues in two adjacent flexible loops were modified to allow for pi-stacking, Fig. 2c. All protein variants and introduced mutations can be seen in Fig. 2d.

### Stability of designed patatins

**Fig. 3:**
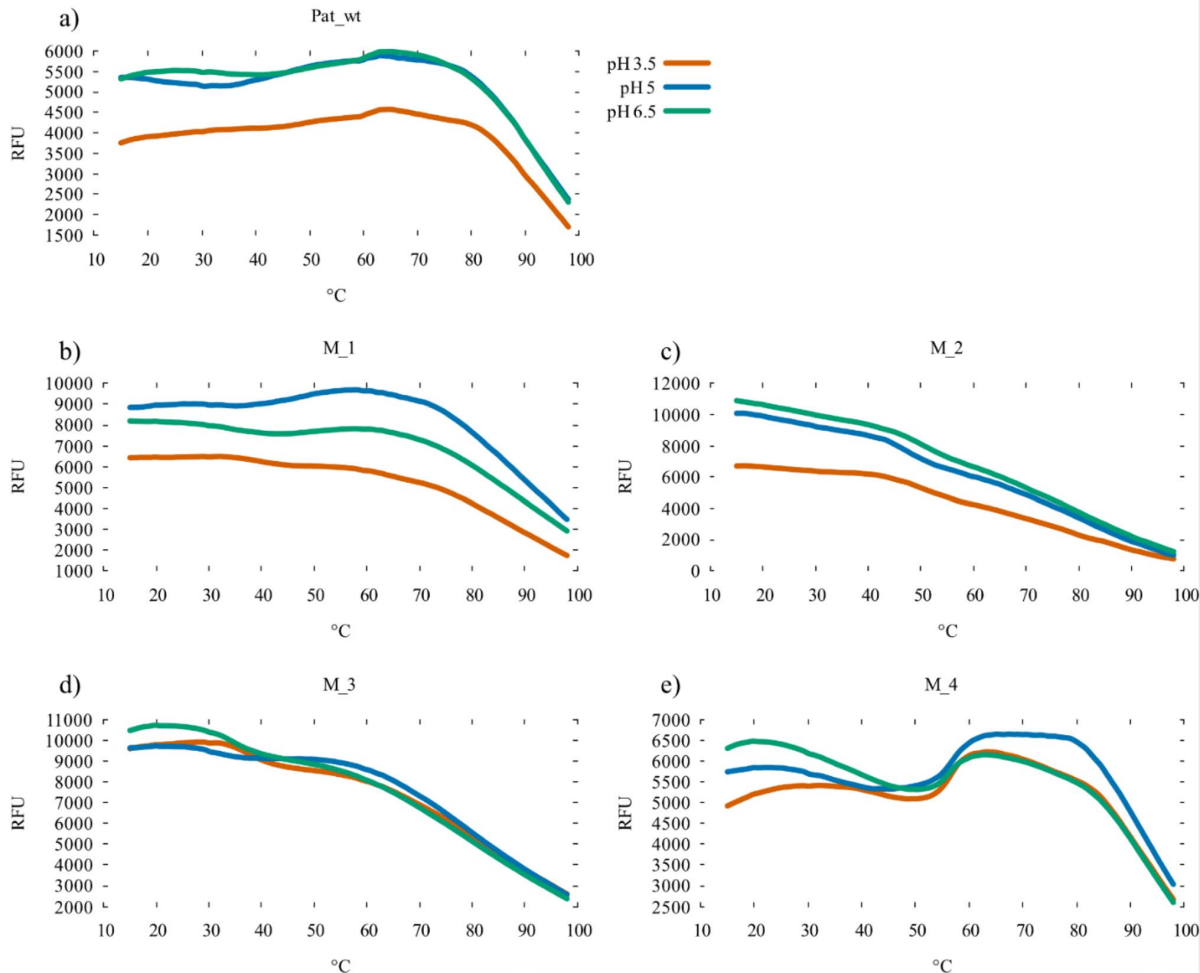
Differential scanning fluorimetry melt curves of recombinant patatin purified from *E. coli*. Each sample was measured at three different pH; 3.5 (orange), pH 5 (blue), and 6.5 (green). Each line represents the mean of four replicates, corrected for background

**Fig. 4:**
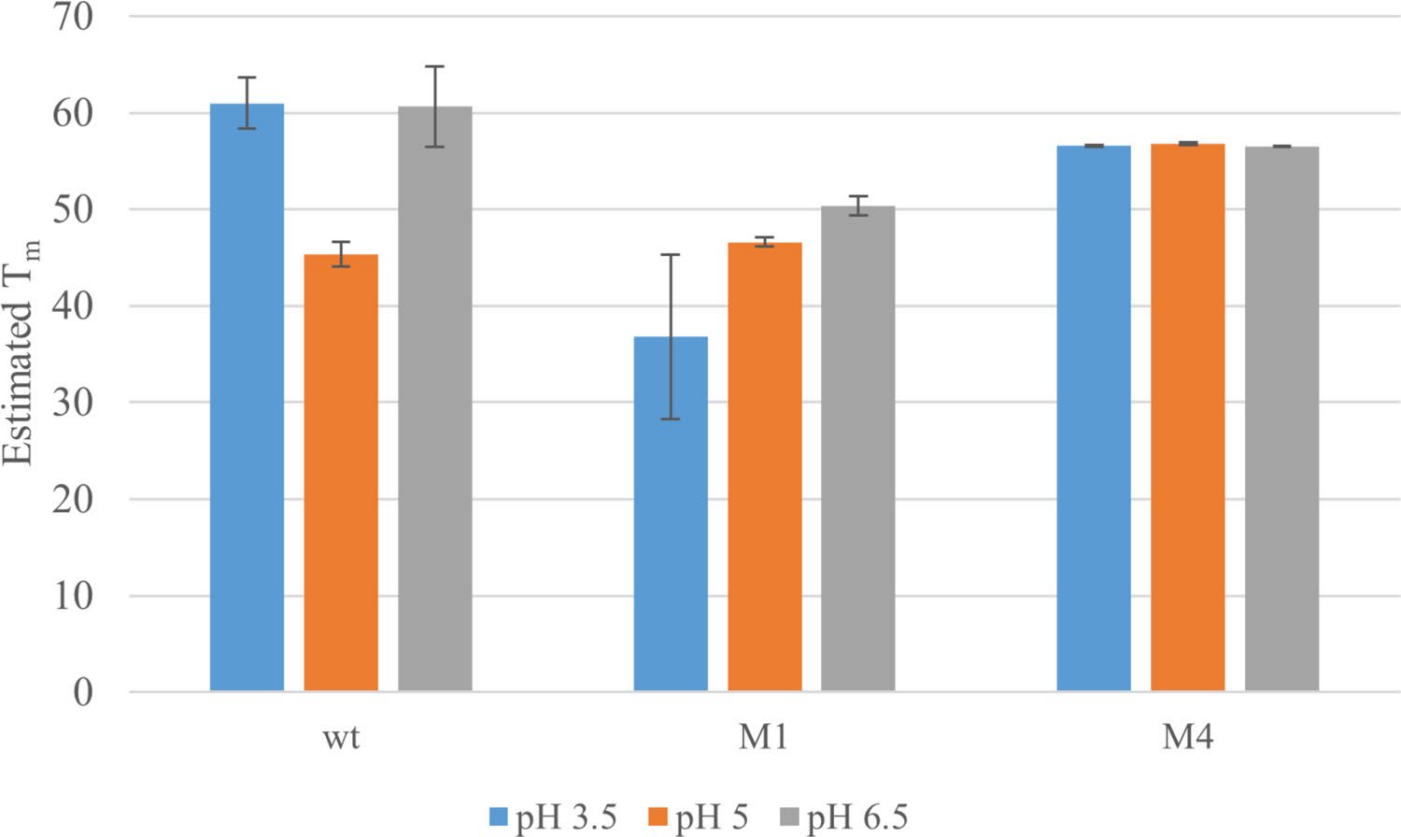
Melting temperature calculated by Boltzmann equation fitting of data from differential scanning fluorimetry. Measurements were done at three different pH; 3.5, 5, and 6.5. Bars represent mean of four replicates and error bars show standard deviation

To investigate the effect of the designed patatin variants carrying mutations, recombinant protein was obtained from heterologous *E. coli* expression as inclusion bodies. The four patatin protein variants were named M1–M4. After refolding, the protein stability was analyzed using differential scanning fluorimetry. Three pH levels were selected for the assay conditions (pH 3.5, pH 5, and pH 6.5) to represent a range found under industrial processing conditions of potato fruit juice (PFJ) to the pH found in potato tubers. The mean of four replicates adjusted for background is presented in Fig. 3. Among the mutant proteins, M1 (Fig. 3b) showed the most wt-like pattern, with a broad peak around 70 °C, and a sharp decrease above 80°C. In contrast, M2 and M3 (Fig. 3c and d, respectively) displayed a more rapid unfolding and aggregate formation than the wild-type protein, and both samples exhibiting high fluorescence already at 15°C. M4 had an interesting profile (Fig. 3e), with two clearly defined peaks, one around 40 °C and a second similar to wt with a plateau around 70°C. Following the initial assessment, differential scanning fluorimetry data were further analyzed using TSA_CRAFT, which utilizes Boltzmann fitting to estimate the proteins melting temperature. With the estimated melting temperature of Pat_wt, M1 and M4 are summarized in Fig. 4. M2 and M3 both failed to provide reliable fitting data. Pat_wt shows a clear decrease of T_m_ at pH 5, which also happens to be close to the proteins pI. In contrast, M1 has increasing T_m_ with increasing pH, while M4 maintains a very even T_m_ at all tested conditions.

## Discussion

With the increasing attention given to plant-based protein products, patatin, a potato tuber storage protein, has the potential to be a valuable side-product from potato starch industry. However, high levels of unhealthy glycoalkaloids in the same side-stream is a serious obstacle in an easy access to this protein source. Due to the high production cost related to the refinement using for example ultrafiltration (Wojnowska et al. 2006; Løkra and Strætkvern 2009), total tuber protein is commonly precipitated using a dual treatment of acid and heat. The resulting protein extract has an unsatisfactory sensory profile and lack economically relevant functional properties such as high foamability (Bártová and Bárta 2008; Løkra and Strætkvern 2009). Biotechnology has been widely applied to solve industrial problems in microbes (Fakhravar and Hesampour 2018), but with the advent of precision genome editing technologies, such as prime editing, there could be an opportunity to design desired outcomes in enzyme or protein functionality in plants.

As a first step in assessing the suitability of the patatin family for gene editing, we analyzed the number of individual patatin genes expressed and the respective levels of expression in tubers from two potato cultivars, Desirée and Kuras. We could determine that the expression patterns differ greatly between cultivars, where differences in observed protein patterns have been reported previously (Bauw et al. 2006; Barta et al. 2012). Out of the 21 gene copies reported by Yang et al. (2023), 19 were found to be present in our studied cultivars. While it is possible that this is purely due to cultivar selection, it could also be an indication that there are still some complications with the assembly of the patatin cluster in DM 8.1. Another interesting finding was that one of the patatin genes in the chromosome 8 cluster was preferentially expressed in Kuras leaves (Supplementary Information SI, Table S2). This is surprising, since this region is thought to house only class-I patatin genes, which are thought to be tuber-specific. Another observation is the prevalence of predicted catalytically inactive patatin variants, both in the genome and among the highly expressed variants. In both investigated cultivars, two out of the three most highly expressed genes carry a S77G mutation, which is known to render the enzyme catalytically inactive. Interestingly, Yang et al. (2023) reported that the patatin cluster in non-tuber carrying relatives, such as eggplant and tomato, is much less complex. This could hint that the origin of the family is lipid-acyl hydrolases, and has later shifted to storage protein in potato. Since the lipase activity could cause adverse side effects, it seems likely that loss of activity and expression of catalytically inactive variants would be preferential to storage proteins.

The investigation of genetic and allelic variation among Desirée and CRISPR/Cas9-mutated events further highlights the importance of CRISPR mutation experiments in deciphering the genetic variation of target genotype. For instance, of the three most common Desirée alleles found in our amplicon sequencing, only one is currently present in NCBI, and the third most common one is missing from DM8.1. Dividing the reads into alleles based on the four nucleotides up-stream of the Cas9 cut site, we can see that the two most common alleles, on a genomic DNA level, can both be found in DM8.1, and are both highly expressed in Kuras and Desirée, with the CAGG allele corresponding to Pat-6, Pat-12, Pat-14, and Pat-15. While the genes Pat-1, Pat-16, and Pat-19 are all CAAG variants. It is also interesting to note that among the highly expressed variants in Desirée, all four of the CAGG type show high expression, while only one (Pat-1) of the CAAG variants has a high expression.

To investigate what impact the underlying genetic variation has on the efficiency of gene editing, CRISPR/Cas9 was used for targeting mutations to the patatin gene family. Amplicon sequencing of a number of putative mutant events showed mutation rates of 6–15%, this corresponds to about two mutant genes per genome. The distribution of edits was further analyzed by comparing the frequency of wild-type alleles to those of the untreated control (Fig. 2d), which showed clear preference for alleles that perfectly matched the sgRNA. In particular, sequence differences to the sgRNA close to the PAM-site were detrimental to functionality in mutagenesis, which has been previously reported (Jinek et al. 2012). While multiplexing for broad targeting, multiplexing for the most highly expressed variants seems necessary. This would also be of great interest, since complete knock-out studies have not, to our knowledge, been done on the patatin family. Although a knock-down study utilizing RNAi showed that removal of patatin does not affect plant growth or phenotype (Kim et al. 2008), it would be interesting to utilize this newfound information to generate full knock-outs of patatin to study what effect it might have on the plants.

With an improved knowledge of the target at hand, we moved on to investigate what modifications to the structure would be required to obtain the desired characteristics for patatin proteins, namely improved thermostability. However, thermostability is known to be a complex trait to modify, and typically, the focus is on improved core packing, increased rigidity, and minimizing surface hydrophobic residues, such as those that can be found in protein-interacting domains (Eijsink et al. 2004; Radestock and Gohlke 2008). When it comes to increasing rigidity, unstructured loops have been of particular interest for modification (Rahban et al. 2022; Thomson et al. 2022). To narrow down the possible targets and permutations, constraint network analysis was used to identify unfolding nuclei. Two areas seem to be of particular interest (Fig. 1a), two of the beta-strands that form the core of the protein, and an alpha helix on the surface. Introduction of di-sulfide bridges has been commonly used to stabilize flexible areas of proteins (Matsumura et al. 1989), and thus, we developed two mutant proteins M1 and M3, with a focus on using cysteines to induce di-sulfide bridges. Both variants showed accelerated aggregation behavior (Fig. 3b and d). This is known to be a potential side effect of using cysteins, since the di-sulfide bridges, if broken up, or not properly formed, can instead form between protein units leading to aggregates forming (Rabdano et al. 2017). It is interesting to note that a recent study showed that cysteines play a small role in patatin aggregate formation when compared to the whey protein β-lactoglobulin (Andlinger et al. 2022). It is therefore possible that increasing the number of free thiol groups in patatin changes the aggregation kinetics. Next, we focused on increasing the number of hydrogen bonds, and on forming salt bridges (Fig. 2b). This led to the design of patatin variant M2, which showed poor stability under all experimental conditions applied. High fluorescence observed already at 15 °C (Fig. 3c) suggests that this mutant protein might not be properly folded, leaving large hydrophobic areas exposed to the surrounding environment. In a fourth patatin variant, a cluster of unstructured loops in close proximity to the unstable alpha helix identified by CNA was targeted. These were stabilized by the introduction of several aromatic amino acids (Fig. 2c), since these are often more prevalent in thermophilic proteins (Eijsink et al. 2004; Modarres et al. 2016), and aromatic clusters have previously been utilized for protein stabilization (Burley and Petsko 1985; Anderson et al. 1993). This mutant protein, M4, showed the largest diversion from wild-type melting behavior (Fig. 3e), with two distinct peaks and a plateau before final aggregation. It has been suggested that patatin unfolds in two steps (Pots et al. 1999), with partial unfolding occurring already at 28°C. The intriguing feature of M4 is the sharp decrease in fluorescence that can be seen between 30 and 50 °C, which suggests that hydrophobic residues are first exposed, then buried, and then exposed again at increasing temperatures. Whether this is due to partial reversible aggregation, or the formation of an intermediary stable conformation of the protein remains to be clarified, but could hold valuable clues for designing stable patatin variants. It has been previously suggested that patatin irreversibly unfolds when treated with acid (Pots et al. 1998b). While this is not evident from our data, it is clear that pH has an effect on both Pat_wt and patatin variant M1. The much lower T_m_ observed at pH 5 coincides with the pI of patatin, which could be an explanation. Patatin variant M1, on the other hand, showed lower T_m_ at lower pH, possibly due to the reduced state of cysteine below pH 5.8. Interestingly, M4 showed incredibly consistent performance across all tested conditions. This is especially interesting for pH 5 where it outperforms Pat_wt. This could make M4 an important starting point for future developments.

A major unknown regarding these designed patatin variants is the effect of glycosylation. While *E. coli* allows for fast turnaround and easy handling, it does not fully allow for post-translational modifications. Since patatin is a known glycoprotein, it could be interesting to further screen mutants using a system which allows for glycosylation. One common example is yeast, which has recently been used to express patatin (Gelley et al. 2022). If this expression could be optimized to allow for library screening, the interaction between mutations and glycosylation could be studied before their introduction into potato patatin genes.

The successful introduction of stabilizing mutations into potato patatin genes would be the ultimate test of the concepts laid out in this study. Unfortunately, the large complexity of the patatin gene cluster identified in this and other studies in combination with the lacklustre efficiency of prime editing in plants (Lin et al. 2020; Perroud et al. 2022) are not an ideal combination. A more efficient gene editing method might be the use of base editors, which have seen a substantial increase in efficiency recently (Westberg et al. 2023). This, however, places greater constraints on what mutations can be successfully introduced, since only some base modifications are currently possible at high enough efficiency. For a targeted approach possibly reducing library screening, there is promising progress in the protein modeling area, with the release of tools, such as AlphaFold2, RoseTTAFold, and ESMFold, the possibilities to do accurate modeling of de-novo designed proteins are improving. These tools have already been applied to design cysteine enriched patatin variants that, when expressed, form polymers with desirable properties for potato flour doughs (Luo et al. 2024). There are plenty of database entries of thermophilic proteins that a model could be trained on (Modarres et al. 2016; Huang et al. 2020), and the model could be configured to focus on mutations that are possible to achieve through the more efficient A-to-G and C-to-T modifications available through base editing.

This type of targeted in-vivo protein design is in its infancy but holds great potential. A challenge with patatin is that it is a cluster of very similar genes and in combination with potato being tetraploid will yield a large number of targets to modify. Thus, improvements of the available techniques are needed, or novel techniques must be developed. However, the presented study can conclude that all patatin genes in the cluster of a specific variety are not equally expressed so there is a great potential for narrowing down the number of targets needed to be modified to reach an impact. A patatin optimized for extractability would be a great addition to the vegetable protein transition due to its high-value nutritional and functional properties and availability from already established industrial side-streams.

## Supplementary Information

Below is the link to the electronic supplementary material.

Supplementary file1 (DOCX 27 KB)

Supplementary file2 (DOCX 31 KB)

## Data Availability

TPM values and protein sequence alignments are included as supplementary material. Additional data are available from the corresponding author on reasonable request.

## Abbreviations

Cas: CRISPR-associated
CAN: Constraint network analysis
CRISPR: Clustered regularly interspaced repeats
HRFA: High-resolution fragment length analysis
RNP: Ribo-nucleo protein
sgRNA: Single-stranded guide RNA
