# Supplementary material for: Modifying the potato tuber storage protein patatin targeting improved thermal stability
Source: Planta. 2025 Jul 11;262(2):46. doi: 10.1007/s00425-025-04766-2 (PMC12254056; doi:10.1007/s00425-025-04766-2)
Supplement: Supplementary file 1 — Supplementary file1 (DOCX 27 KB) [file 425_2025_4766_MOESM1_ESM.docx]

**Modifying potato tuber storage protein Patatin for improved thermal stability and functionality**

Planta

Martin Friberg*, Shrikant Sharma, Folke Sitbon, Mariette Andersson, Per Hofvander
* Corresponding author, Swedish University of Agriculture, Department of Plant Breeding, martin.friberg@slu.se

# **Supplementary Information SI:** TPM-values for mapping Desirée and Kuras expression data to the DM 8.1 genome.

## **Table S1** Tuber expression, TPM values before averaging

|  | Desirée.1 | Desirée.2 | Desirée.3 | Kuras.1 | Kuras.2 | Kuras.3 |
| --- | --- | --- | --- | --- | --- | --- |
| DM8C08G01480 | 3006.12 | 3007.64 | 464.57 | 518.37 | 804.05 | 650.60 |
| DM8C08G01490 | 139.77 | 163.12 | 59.48 | 977.31 | 1265.51 | 992.46 |
| DM8C08G01500 | 0.00 | 0.00 | 0.00 | 0.00 | 0.00 | 0.00 |
| DM8C08G01510 | 6.55 | 4.23 | 0.98 | 0.98 | 0.97 | 0.77 |
| DM8C08G01530 | 0.24 | 0.65 | 0.00 | 0.13 | 0.00 | 0.12 |
| DM8C08G01540 | 5718.59 | 6072.75 | 688.54 | 463.74 | 476.99 | 360.64 |
| DM8C08G01550 | 3.85 | 0.88 | 0.00 | 0.00 | 0.00 | 0.00 |
| DM8C08G01560 | 0.00 | 0.00 | 0.00 | 0.00 | 0.00 | 0.00 |
| DM8C08G01570 | 505.59 | 550.98 | 157.77 | 7.97 | 4.95 | 6.81 |
| DM8C08G01580 | 0.52 | 0.00 | 0.00 | 0.00 | 0.00 | 0.00 |
| DM8C08G01590 | 0.39 | 0.77 | 0.43 | 12.55 | 20.90 | 12.83 |
| DM8C08G01600 | 1244.37 | 1271.37 | 125.47 | 62.66 | 68.20 | 59.85 |
| DM8C08G01620 | 26.22 | 21.35 | 2.34 | 0.00 | 0.00 | 0.00 |
| DM8C08G01630 | 2313.55 | 2451.82 | 506.76 | 142.79 | 129.62 | 107.45 |
| DM8C08G01640 | 1071.21 | 1160.97 | 76.95 | 31.54 | 42.45 | 28.54 |
| DM8C08G01650 | 66.82 | 77.12 | 28.39 | 830.47 | 1061.72 | 813.12 |
| DM8C08G01660 | 0.00 | 0.00 | 0.00 | 0.00 | 0.34 | 0.00 |
| DM8C08G01670 | 0.00 | 0.04 | 0.02 | 2.48 | 2.29 | 2.07 |
| DM8C08G01690 | 0.00 | 0.00 | 0.00 | 2.73 | 4.69 | 2.71 |

## **Table S2** Leaf expression, TPM values before averaging

|  | Desirée.1 | Desirée.2 | Desirée.3 | Kuras.1 | Kuras.2 | Kuras.3 |
| --- | --- | --- | --- | --- | --- | --- |
| DM8C08G01480 | 0.00 | 0.04 | 0.69 | 2.07 | 5.37 | 0.55 |
| DM8C08G01490 | 2.07 | 4.10 | 16.58 | 5.04 | 22.93 | 2.24 |
| DM8C08G01500 | 0.00 | 0.00 | 0.00 | 0.00 | 0.00 | 0.00 |
| DM8C08G01510 | 0.00 | 0.00 | 0.00 | 0.00 | 0.00 | 0.00 |
| DM8C08G01530 | 0.00 | 0.00 | 0.00 | 0.00 | 0.00 | 0.00 |
| DM8C08G01540 | 0.09 | 0.14 | 0.45 | 19.18 | 4.06 | 2.25 |
| DM8C08G01550 | 0.00 | 0.00 | 0.00 | 0.00 | 0.00 | 0.00 |
| DM8C08G01560 | 0.00 | 0.00 | 0.00 | 0.00 | 0.00 | 0.00 |
| DM8C08G01570 | 0.00 | 0.00 | 0.00 | 2.94 | 0.00 | 0.00 |
| DM8C08G01580 | 0.00 | 0.00 | 0.00 | 0.00 | 0.00 | 0.00 |
| DM8C08G01590 | 0.00 | 0.00 | 0.00 | 0.00 | 0.00 | 0.00 |
| DM8C08G01600 | 0.00 | 0.00 | 0.00 | 7.27 | 9.04 | 0.62 |
| DM8C08G01620 | 0.00 | 0.00 | 0.00 | 0.00 | 0.26 | 0.06 |
| DM8C08G01630 | 0.00 | 0.00 | 0.00 | 4.50 | 0.00 | 0.26 |
| DM8C08G01640 | 0.09 | 0.14 | 0.45 | 0.92 | 0.00 | 0.00 |
| DM8C08G01650 | 0.41 | 0.46 | 1.30 | 2.39 | 9.32 | 1.68 |
| DM8C08G01660 | 0.00 | 0.00 | 0.00 | 0.00 | 0.00 | 0.00 |
| DM8C08G01670 | 1.58 | 1.61 | 2.36 | 0.94 | 2.30 | 0.95 |
| DM8C08G01690 | 1.84 | 1.84 | 1.35 | 138.57 | 282.96 | 106.50 |
