# Supplementary material for: Modifying the potato tuber storage protein patatin targeting improved thermal stability
Source: Planta. 2025 Jul 11;262(2):46. doi: 10.1007/s00425-025-04766-2 (PMC12254056; doi:10.1007/s00425-025-04766-2)
Supplement: Supplementary file 2 — Supplementary file2 (DOCX 31 KB) [file 425_2025_4766_MOESM2_ESM.docx]

**Modifying potato tuber storage protein Patatin for improved thermal stability and functionality**

Planta

Martin Friberg*, Shrikant Sharma, Folke Sitbon, Mariette Andersson, Per Hofvander
* Corresponding author, Swedish University of Agriculture, Department of Plant Breeding, martin.friberg@slu.se

# **Supplementary Information SII**: **Patatin protein sequence length and alignment**

## Protein length of patatin genes studied. Names refer to the published gene or transcript

NP_001275345 387 aa
DM8C08G01480.1 375 aa
DQ114415 387 aa
DQ114416 387 aa
DM8C08G01490.1 385 aa
DM8C08G01490.1.2 385 aa
DQ114417 386 aa
DQ114418 386 aa
DQ114419 386 aa
DQ114420 386 aa
DM8C08G01650.1 335 aa
DM8C08G01650.1.2 335 aa
DQ114421 386 aa
DM8C08G01540.2 386 aa
DM8C08G01540.2.2 386 aa
X03932 386 aa
DM8C08G01640.2 386 aa
DM8C08G01640.2.2 386 aa
DM8C08G01600.1 346 aa
DM8C08G01600.1.2 346 aa
DM8C08G01630.1 332 aa
DM8C08G01630.1.2 332 aa
DM8C08G01690.1 360 aa
DM8C08G01690.1.2 360 aa
DM8C08G01500.1 99 aa
DM8C08G01510.1 219 aa
DM8C08G01530.1 206 aa
DM8C08G01550.1 110 aa
DM8C08G01560.1 72 aa
DM8C08G01570.1 156 aa
DM8C08G01580.1 110 aa
DM8C08G01590.1 158 aa
DM8C08G01620.1 204 aa
DM8C08G01660.1 264 aa
DM8C08G01670.1 478 aa

## ClustalW sequence alignment of patatin amino acid sequences

DM8C08G01650.1 ------------------------------------------------------------

DM8C08G01650.1.2 ------------------------------------------------------------

DM8C08G01480.1 ------------------------------------------------------------

DQ114415 ------------------------------------------------------------

NP_001275345 ------------------------------------------------------------

DQ114416 ------------------------------------------------------------

DM8C08G01660.1 ------------------------------------------------------------

DM8C08G01640.2 ------------------------------------------------------------

DM8C08G01500.1 ------------------------------------------------------------

DM8C08G01640.2.2 ------------------------------------------------------------

DM8C08G01630.1 ------------------------------------------------------------

DM8C08G01630.1.2 ------------------------------------------------------------

DQ114421 ------------------------------------------------------------

DM8C08G01540.2 ------------------------------------------------------------

DM8C08G01540.2.2 ------------------------------------------------------------

DM8C08G01550.1 ------------------------------------------------------------

DM8C08G01570.1 ------------------------------------------------------------

DM8C08G01580.1 ------------------------------------------------------------

DM8C08G01600.1 ------------------------------------------------------------

DM8C08G01600.1.2 ------------------------------------------------------------

DM8C08G01490.1 ------------------------------------------------------------

DM8C08G01490.1.2 ------------------------------------------------------------

DM8C08G01590.1 ------------------------------------------------------------

DM8C08G01670.1 MTILMAFQTPEVEVIGLATIFGNVTTKDATRNALLLLLNTLSPNLTLVSCYRGEPCVVDF

DM8C08G01560.1 ------------------------------------------------------------

DM8C08G01530.1 ------------------------------------------------------------

DM8C08G01690.1 ------------------------------------------------------------

DM8C08G01690.1.2 ------------------------------------------------------------

X03932 ------------------------------------------------------------

DQ114417 ------------------------------------------------------------

DQ114419 ------------------------------------------------------------

DQ114418 ------------------------------------------------------------

DQ114420 ------------------------------------------------------------

DM8C08G01510.1 ------------------------------------------------------------

DM8C08G01620.1 ------------------------------------------------------------

DM8C08G01650.1 ------------------------------------------------------------

DM8C08G01650.1.2 ------------------------------------------------------------

DM8C08G01480.1 ------------------------------------------------------------

DQ114415 ------------------------------------------------------------

NP_001275345 ------------------------------------------------------------

DQ114416 ------------------------------------------------------------

DM8C08G01660.1 ------------------------------------------------------------

DM8C08G01640.2 ------------------------------------------------------------

DM8C08G01500.1 ------------------------------------------------------------

DM8C08G01640.2.2 ------------------------------------------------------------

DM8C08G01630.1 ------------------------------------------------------------

DM8C08G01630.1.2 ------------------------------------------------------------

DQ114421 ------------------------------------------------------------

DM8C08G01540.2 ------------------------------------------------------------

DM8C08G01540.2.2 ------------------------------------------------------------

DM8C08G01550.1 ------------------------------------------------------------

DM8C08G01570.1 ------------------------------------------------------------

DM8C08G01580.1 ------------------------------------------------------------

DM8C08G01600.1 ------------------------------------------------------------

DM8C08G01600.1.2 ------------------------------------------------------------

DM8C08G01490.1 ------------------------------------------------------------

DM8C08G01490.1.2 ------------------------------------------------------------

DM8C08G01590.1 ------------------------------------------------------------

DM8C08G01670.1 VHGSVGLGNLFLPSPNSKKIDKSVSEFLVEKVSEYPGEMSILALRPLTNLALAVKWDSTF

DM8C08G01560.1 ------------------------------------------------------------

DM8C08G01530.1 ------------------------------------------------------------

DM8C08G01690.1 ------------------------------------------------------------

DM8C08G01690.1.2 ------------------------------------------------------------

X03932 ------------------------------------------------------------

DQ114417 ------------------------------------------------------------

DQ114419 ------------------------------------------------------------

DQ114418 ------------------------------------------------------------

DQ114420 ------------------------------------------------------------

DM8C08G01510.1 ------------------------------------------------------------

DM8C08G01620.1 ------------------------------------------------------------

DM8C08G01650.1 ------------------------------------------------------------

DM8C08G01650.1.2 ------------------------------------------------------------

DM8C08G01480.1 ------------------------------------------------------------

DQ114415 ------------------------------------------------------------

NP_001275345 ------------------------------------------------------------

DQ114416 ------------------------------------------------------------

DM8C08G01660.1 ------------------------------------------------------------

DM8C08G01640.2 ------------------------------------------------------------

DM8C08G01500.1 ------------------------------------------------------------

DM8C08G01640.2.2 ------------------------------------------------------------

DM8C08G01630.1 ------------------------------------------------------------

DM8C08G01630.1.2 ------------------------------------------------------------

DQ114421 ------------------------------------------------------------

DM8C08G01540.2 ------------------------------------------------------------

DM8C08G01540.2.2 ------------------------------------------------------------

DM8C08G01550.1 ------------------------------------------------------------

DM8C08G01570.1 ------------------------------------------------------------

DM8C08G01580.1 ------------------------------------------------------------

DM8C08G01600.1 ------------------------------------------------------------

DM8C08G01600.1.2 ------------------------------------------------------------

DM8C08G01490.1 ------------------------------------------------------------

DM8C08G01490.1.2 ------------------------------------------------------------

DM8C08G01590.1 ------------------------------------------------------------

DM8C08G01670.1 ASKVKRVVVLGDSFFAVENVNPAAQANVGINITTQVQLKDADLEELKQSEGKHANNTTVR

DM8C08G01560.1 ------------------------------------------------------------

DM8C08G01530.1 ------------------------------------------------------------

DM8C08G01690.1 ------------------------------------------------------------

DM8C08G01690.1.2 ------------------------------------------------------------

X03932 ------------------------------------------------------------

DQ114417 ------------------------------------------------------------

DQ114419 ------------------------------------------------------------

DQ114418 ------------------------------------------------------------

DQ114420 ------------------------------------------------------------

DM8C08G01510.1 ------------------------------------------------------------

DM8C08G01620.1 ------------------------------------------------------------

DM8C08G01650.1 ------------------------------------------------MILATTSSTCAK

DM8C08G01650.1.2 ------------------------------------------------MILATTSSTCAK

DM8C08G01480.1 ------------------------------------------------MILATTSSTFAT

DQ114415 ------------------------------------MATTKSVLVLIFMILATTSSTFAT

NP_001275345 ------------------------------------MATTKSVLVLIFMILATTSSTFAT

DQ114416 ------------------------------------MATTKSVLVLIFMILATTSSTFAS

DM8C08G01660.1 ------------------------------------------------MLLATTSSTLAS

DM8C08G01640.2 ------------------------------------MATTKSFTILIFMMLATTSSTFAT

DM8C08G01500.1 ------------------------------------------------------------

DM8C08G01640.2.2 ------------------------------------MATTKSFTILIFMMLATTSSTFAT

DM8C08G01630.1 ------------------------------------------------------------

DM8C08G01630.1.2 ------------------------------------------------------------

DQ114421 ------------------------------------MATTKYFTILIFMMLATTSSTFAT

DM8C08G01540.2 ------------------------------------MATTKYFTILIFMMLATTSSTFAT

DM8C08G01540.2.2 ------------------------------------MATTKYFTILIFMMLATTSSTFAT

DM8C08G01550.1 ------------------------------------------------------------

DM8C08G01570.1 ------------------------------------------------------------

DM8C08G01580.1 ------------------------------------------------------------

DM8C08G01600.1 ------------------------------------------------------------

DM8C08G01600.1.2 ------------------------------------------------------------

DM8C08G01490.1 ----------MKPMRKWTIIQDAR-------------------------LADYFDVIGGT

DM8C08G01490.1.2 ----------MKPMRKWTIIQDAR-------------------------LADYFDVIGGT

DM8C08G01590.1 ------------------------------------------------------------

DM8C08G01670.1 LIDTCLCKAAMTPTPPFGLIRTIQGWLTCFYFIELQCVQLYLSEMAPYKYRDALNTTGGV

DM8C08G01560.1 ------------------------------------------------------------

DM8C08G01530.1 ------------------------------------------------------------

DM8C08G01690.1 ------------------------------------------------------------

DM8C08G01690.1.2 ------------------------------------------------------------

X03932 ------------------------------------MATTNSFTILIFMILATTSSTFAT

DQ114417 ------------------------------------MATTKSVLVLIFMILATTGSTCAT

DQ114419 ------------------------------------MATTKSVLVLFFMILATTSSTCAT

DQ114418 ------------------------------------MATTKSFLILFFMILATTSSTCAT

DQ114420 ------------------------------------MATTKSFLILFFMILATTSSTCAT

DM8C08G01510.1 ------------------------------------------------------------

DM8C08G01620.1 ------------------------------------------------------------

DM8C08G01650.1 LEEMVTVLSIDGGGIKGIIPATILEFLEGQLQEVDNNKDARLADYFDVIGGTSTGGLLTA

DM8C08G01650.1.2 LEEMVTVLSIDGGGIKGIIPATILEFLEGQLQEVDNNKDARLADYFDVIGGTSTGGLLTA

DM8C08G01480.1 LGEMVTVLSIDGGGIKGIIPGIILEFLEGQLQKMDNNADARLADYFDVIGGTSTGGLLTA

DQ114415 LGEMVTVLSIDGGGIKGIIPGIILEFLEGQLQKMDNNADARLADYFDVIGGTSTGGLLTA

NP_001275345 LGEMVTVLSVDGGGIKGIIPGIILEFLEGQLQKMDNNADARLADYFDVIGGTSTGGLLTA

DQ114416 LEEMVTVLSIDGGGIKGIIPGTILEFLEGQLQKMDNNADARLADYFDVIGGTSTGGLLTA

DM8C08G01660.1 LEEMVTVLSIDGGAIRGIIPGVILEFLEGELQRIDNNPDARLADYFDVIGGTSTGGLLAA

DM8C08G01640.2 LGEMVTVLSIDGGGIKGIIPATILEFLEGQLQEVDNNTDARLADYFDVIGGTGTGGLLTA

DM8C08G01500.1 ------------------------------------------------------------

DM8C08G01640.2.2 LGEMVTVLSIDGGGIKGIIPATILEFLEGQLQEVDNNTDARLADYFDVIGGTGTGGLLTA

DM8C08G01630.1 ---MVTVLSIDGGGIKGIIPATILEFLEGQLQEVDNNTDARLADYFDVIGGTGTGGLLTA

DM8C08G01630.1.2 ---MVTVLSIDGGGIKGIIPATILEFLEGQLQEVDNNTDARLADYFDVIGGTGTGGLLTA

DQ114421 LGEMVTVLSIDGGGIKGIIPATILEFLEGQLQEVDNNTDARLADYFDVIGGTGTGGLLTA

DM8C08G01540.2 LGEMVTVLSIDGGGIKGIIPATILEFLEGQLQEVDNNTDARLADYFDVIGGTGTGGLLTA

DM8C08G01540.2.2 LGEMVTVLSIDGGGIKGIIPATILEFLEGQLQEVDNNTDARLADYFDVIGGTGTGGLLTA

DM8C08G01550.1 ------------------------------------------------------------

DM8C08G01570.1 ------------------------------------------------------------

DM8C08G01580.1 ------------------------------------------------------------

DM8C08G01600.1 ----------------MISCGSAMSRKEGQLQEVDNNTDARLADYFDVIGGTGTGGLLTA

DM8C08G01600.1.2 ----------------MISCGSAMSRKEGQLQEVDNNTDARLADYFDVIGGTGTGGLLTA

DM8C08G01490.1 STGGLLTAMITTPNENNRPFVAAKDIVPFYFQHG--PMIFEFIDYFDVIGGTSTGGLLTA

DM8C08G01490.1.2 STGGLLTAMITTPNENNRPFVAAKDIVPFYFQHG--PMIFEFIDYFDVIGGTSTGGLLTA

DM8C08G01590.1 ------------------------------------------------------------

DM8C08G01670.1 NVIAAVVSIYYVDKLRMRLLFLEGGVQMLFSQDVDKNTDARLADYFDVIGGTSTGGLLAA

DM8C08G01560.1 ------------------------------------------------------------

DM8C08G01530.1 ------------------------------------------------------------

DM8C08G01690.1 ---MVTVLSIDGGGIKGIIPGTILEFLEGQLQDVDNNKDARLADYFDVIGGTSTGGLLTA

DM8C08G01690.1.2 ---MVTVLSIDGGGIKGIIPGTILEFLEGQLQDVDNNKDARLADYFDVIGGTSTGGLLTA

X03932 LGEMVTVLSIDGGGIKGIIPATILEFLEGQLQEVDNNTDARLADYFDVIGGTSTGGLLTA

DQ114417 LGEMVTVLSIDGGGIKGIIPATILEFLEGQLQEVDNNKDARLADYFDVIGGTSTGGLLTA

DQ114419 LGEMVTVLSIDGGGIKGIIPATILEFLEGQLQEVDNNKDARLADYFDVIGGTSTGGLLTA

DQ114418 LGEMVTVLSIDGGGIKGIIPAVILEFLEGQLQEVDNNKDARLADYFDVIGGTSTGGLLTA

DQ114420 LGEMVTVLSIDGGGIKGIIPAIILEFLEGQLQEVDNNADARLADYFDVIGGTSTGGLLTA

DM8C08G01510.1 ------------------------------MKTIDPLLLLKILYLFTSIMALRFLNLVVF

DM8C08G01620.1 ---MVTVLSIDGGGIKGIIPATILEFLEGQLQEVDNNTDARLADYFDVIGGTVREVYPLL

DM8C08G01650.1 MITTPNENNRPFAAAKDIVPFYFEHGPHIFNS---SGSIFGPRYDGKYLLQVLQEKLGET

DM8C08G01650.1.2 MITTPNENNRPFAAAKDIVPFYFEHGPHIFNS---SGSIFGPRYDGKYLLQVLQEKLGET

DM8C08G01480.1 MITTPNENNRPFAAAKDIVPFYFQHGPHIFN--SSTGQFFGPKYDGKYLMQVLQEKLGET

DQ114415 MITTPNENNRPFAAAKDIVPFYFQHGPHIFN--SSTGQFFGPKYDGKYLMQVLQEKLGET

NP_001275345 MITTPNENNRPFAAAKDIVPFYFQHGPHIFN--SSTGQFFGPKYDGKYLMQVPQEKLGET

DQ114416 MITTPNENNRPFAAANEIVPFYFEHGPHIFN--SSTGQFFGPKYDGKYLMQVLQEKLGET

DM8C08G01660.1 MITTPNENNRPFAAANEIVPFYFEHGPSIFSPRPLFPIIPGPKYNSTYLMQVIKEKMGET

DM8C08G01640.2 MITTPNENNRPFAAAKDIIPFYFDHGPKIFEP--SGFHLVEPKYDGKYLMQVLQEKLGET

DM8C08G01500.1 MITTPNENNRPFAAAKDIIPFYFDHGPKIFEP--SGFHLFEPKYDGKYLMQVLQEKL---

DM8C08G01640.2.2 MITTPNENNRPFAAAKDIIPFYFDHGPKIFEP--SGFHLVEPKYDGKYLMQVLQEKLGET

DM8C08G01630.1 MITTPNENNRPFAAAKDIIPFYFDHGPKIFEP--SGFHLVEPKYDGKYLMQVLQEKLGET

DM8C08G01630.1.2 MITTPNENNRPFAAAKDIIPFYFDHGPKIFEP--SGFHLVEPKYDGKYLMQVLQEKLGET

DQ114421 MITTPNENNRPFAAAKDIIPFYFDHGPKIFEP--SGFHLVEPKYDGKYLMQVLQEKLGET

DM8C08G01540.2 MITTPNENNRPFAAAKDIIPFYFDHGPKIFEP--SGFHLVEPKYDGKYLMQVLQEKLGET

DM8C08G01540.2.2 MITTPNENNRPFAAAKDIIPFYFDHGPKIFEP--SGFHLVEPKYDGKYLMQVLQEKLGET

DM8C08G01550.1 ------------------------------------------------------------

DM8C08G01570.1 ------------------------------------------------------------

DM8C08G01580.1 ------------------------------------------------------------

DM8C08G01600.1 MITTPNENNRPFAAAKDIIPFYFDHGPKIFEP--SGFHLVEPKYDGKYLMQVLQEKLGET

DM8C08G01600.1.2 MITTPNENNRPFAAAKDIIPFYFDHGPKIFEP--SGFHLVEPKYDGKYLMQVLQEKLGET

DM8C08G01490.1 IITTPNENNRPFAAAKDIVPFYFEHGPHIFN---NSGSIFGPKYDGKYLMQVLQEKLGET

DM8C08G01490.1.2 IITTPNENNRPFAAAKDIVPFYFEHGPHIFN---NSGSIFGPKYDGKYLMQVLQEKLGET

DM8C08G01590.1 MITTPNGNNRPFAAAKDIIPFYFDHGPKIFEP--SGFHLFEPKYDGKYLMQVLQEKLGET

DM8C08G01670.1 MITTPNETNRPFAAAKDIAPFYFEHGPKIFQ---SSGQNSGPIYDGKYLMHVLQEKFGET

DM8C08G01560.1 MITTPNENNRPFVFAKDIIPFYFDHGPKIFEP--SGFHLVEPKYDGKYLMQVLQEKLGET

DM8C08G01530.1 MITTPNENNRPFAVAKDIIPFYFDHGPKIFEP--SGFHLFEPKYDGKYLMQVLQETLGET

DM8C08G01690.1 MITTPNENNRPFAAAKDIVPFYFKYGPHIFNS--SDPQIFGPINDGKYFMQVLQEKLGET

DM8C08G01690.1.2 MITTPNENNRPFAAAKDIVPFYFKYGPHIFNS--SDPQIFGPINDGKYFMQVLQEKLGET

X03932 MITTPNETNRPFAAAKDIVPFYFEHGPKIFQ---SSGSIFGPKYDGKYLMQVLQEKLGET

DQ114417 MITTPNENNRPFAAAKDIVPFYFEHGPHIFN---SSGTIFGPMYDGKYLLQVLQEKLGET

DQ114419 MITTPNENNRPFAAAKDIVPFYFEHGPHIFN---SSGSIFGPMYDGKYFLQVLQEKLGET

DQ114418 MITTPNENNRPFAAAKDIIPFYFEHGPHIFN---YSGSIFGPMYDGKYLLQVLQEKLGET

DQ114420 MITTPNENNRPFAAAKDIVPFYFEHGPHIFN---YSGSILGPMYDGKYLLQVLQEKLGET

DM8C08G01510.1 TFLSQN------MMENILCKFFKKHLEKLVCIK-----LQKLPSQALTSKRISQYSLSQI

DM8C08G01620.1 LQMKTIDPLQPLKILYLLLRSWPDFTWFSPCA---------KIWKISYAISKNSRIGCDV

DM8C08G01650.1 RVHQALTEVAISSFDIKTNKPVIFTKSNLAKSPELDAKMYDICTLLLILVMVIYMSSILL

DM8C08G01650.1.2 RVHQALTEVAISSFDIKTNKPVIFTKSNLAKSPELDAKMYDICTLLLILVMVIYMSSILL

DM8C08G01480.1 RVHQALTEVAISSFDIKTNKPVIFTKSNLAKSPELDAKMSDICYSTAAAPTYFPPHYFAT

DQ114415 RVHQALTEVAISSFDIKTNKPVIFTKSNLAKSPELDAKMSDICYSTAAAPTYFPPHYFAT

NP_001275345 RVHQALTEVAISSFDIKTNKPVIFTKSNLAKSPELDAKMSDICYSTAAAPTYFPPHYFAT

DQ114416 RVHQALTEVAISSFDIKTNKPVIFTKSNLAKSPELDAKMYDICYSTAAAPTYFPPHYFAT

DM8C08G01660.1 RLNEALTEVVLSTFDIKTNKPVIFTKSSLAESPELNAKMYDICYSTAAAPTYFAPHYFAT

DM8C08G01640.2 RVHQALTEVAISSFDIKTNKPVIFTKSNLAKTPELDAKMYDICYSTAAAPTYFPPHYFAT

DM8C08G01500.1 ------------------------------------------------------------

DM8C08G01640.2.2 RVHQALTEVAISSFDIKTNKPVIFTKSNLAKTPELDAKMYDICYSTAAAPTYFPPHYFAT

DM8C08G01630.1 RVHQALTEVAISSFDIKTNKPVIFTKSNLAKTPELDAKMYDICYSTAAAPTYFPPHYFAT

DM8C08G01630.1.2 RVHQALTEVAISSFDIKTNKPVIFTKSNLAKTPELDAKMYDICYSTAAAPTYFPPHYFAT

DQ114421 RVHQALTEVAISSFDIKTNKPVIFTKSNLAKTPELDAKMYDICYSTAAAPTYFPPHYFAT

DM8C08G01540.2 RVHQALTEVAISSFDIKTNKPVIFTKSNLAKTPELDAKMYDICYSTAAAPTYFPPHYFAT

DM8C08G01540.2.2 RVHQALTEVAISSFDIKTNKPVIFTKSNLAKTPELDAKMYDICYSTAAAPTYFPPHYFAT

DM8C08G01550.1 --------------------------------------MYDICYSTAAAPTYFPPHYFAT

DM8C08G01570.1 --------------------------------------MYDICYSTAAAPTYFPPHYFAT

DM8C08G01580.1 --------------------------------------MYDICYSTAAAPTYFPPHYFAT

DM8C08G01600.1 RVHQALTEVAISSFDIKTNKPVIFTKSNLAKTPELDAKMYDICYSTAAAPTYFPPHYFAT

DM8C08G01600.1.2 RVHQALTEVAISSFDIKTNKPVIFTKSNLAKTPELDAKMYDICYSTAAAPTYFPPHYFAT

DM8C08G01490.1 RVHQALTEVAISSFDIKTNKPVIFTKSNLANAPELDAKMYDICYSTAAAPIYFPPHYFTT

DM8C08G01490.1.2 RVHQALTEVAISSFDIKTNKPVIFTKSNLANAPELDAKMYDICYSTAAAPIYFPPHYFTT

DM8C08G01590.1 RVHQALTEVAISSFDIKTNKPVIFTKSNLAKTPELDAKMYDICYSTAAAPTYFPPHYFAT

DM8C08G01670.1 RLHQALTEVAISTFDIKTNKPVIFTKSQLAKSPELDAKMYDISYSTAAAPVYFPPHYFVT

DM8C08G01560.1 RVHQALTEVAISSF----------------------------------------------

DM8C08G01530.1 RVHQALTEVAISSFDIKTNKPVIFTKSNLAKTPELDAKMYDICYSTAAAPTYFPPHYFAT

DM8C08G01690.1 RLHQALTEVAISTFDIKTNKPVIFTKSQLAKSPELDAKMYDICYSTAAVPMYFPPHYFVT

DM8C08G01690.1.2 RLHQALTEVAISTFDIKTNKPVIFTKSQLAKSPELDAKMYDICYSTAAVPMYFPPHYFVT

X03932 RVHQALTEVAISSFDIKTNKPVIFTKSNLAKSPELDAKMYDICYSTAAAPTFFPPHYFAT

DQ114417 RVHQALTEVAISSFDIKTNKPVIFTKSNLAKSPELDAKMYDICYSTAAAPIYFPPHYFVT

DQ114419 RVHQALTEVAISSFDIKTNKPVIFTKSNLAKSPELDAKMYDICYSTAAAPTYFPPHYFVT

DQ114418 RVHQALTEVAISSFDIKTNKPVIFTKSNLAKSPELDAKMYDICYSTAAAPMYFPPHYFIT

DQ114420 RVHQALTEVAISSFDIKTNKPVIFTKSNLAESPQLDAKMYDICYSTAAAPIYFPPHYFVT

DM8C08G01510.1 QKLQNWMLRCMTYVIPQQQLQHIFLHITLLLILVMEINMTSILLMAMLLLLIRRYYPLAL

DM8C08G01620.1 HMLFHSSSSNIFSSTLLCYYWRSILQSCWRCCCCSGKCINSILLHLMMLLWLIYYYKLVK

DM8C08G01650.1 MVVLLLLVIRRYYPLALQRDLHK-------------------------------------

DM8C08G01650.1.2 MVVLLLLVIRRYYPLALQRDLHK-------------------------------------

DM8C08G01480.1 NTSNGDKYEFNLVDGAVATVADPALLSVSVATRRAEEDPAFASIRSLNYKQLLLLSLGTG

DQ114415 NTSNGDKYEFNLVDGAVATVADPALLSVSVATRRAEEDPAFASIRSLNYKQLLLLSLGTG

NP_001275345 NTSNGDKYEFNLVDGAVATVADPALLSVSVATRRAEEDPAFASIRSLNYKQLLLLSLGTG

DQ114416 NTINGDKYKFNLVDGAVATVADPALLSVSVATRRAQEDPAFASIRSLNYKKMLLLSLGTG

DM8C08G01660.1 TTSNGDPYEFNLVDGGVATVGS--------------------------------------

DM8C08G01640.2 NTSNGDQYDFNLVDGDVAAVDPS-LLSISVATRLAQEDPAFASIKSLNYKQMLLLSLGTG

DM8C08G01500.1 ------------------------------------------------------------

DM8C08G01640.2.2 NTSNGDQYDFNLVDGDVAAVDPS-LLSISVATRLAQEDPAFASIKSLNYKQMLLLSLGTG

DM8C08G01630.1 NTSNGDQYDFNLVDGDVAAVDPS-LLSISVATRLAQEDPAFASIKSLNYKQMLLLSLGTG

DM8C08G01630.1.2 NTSNGDQYDFNLVDGDVAAVDPS-LLSISVATRLAQEDPAFASIKSLNYKQMLLLSLGTG

DQ114421 NTSNGDQYDFNLVDGDVAAVDPS-LLSISVATRLAQEDPAFASIKSLNYKQMLLLSLGTG

DM8C08G01540.2 NTSNGDQYDFNLVDGDVAAVDPS-LLSISVATRLAQEDPAFASIKSLNYKQMLLLSLGTG

DM8C08G01540.2.2 NTSNGDQYDFNLVDGDVAAVDPS-LLSISVATRLAQEDPAFASIKSLNYKQMLLLSLGTG

DM8C08G01550.1 NTSNGDQYDFNLVDGDVAAVDP--------------------------------------

DM8C08G01570.1 NTSNGDQYDFNLVDGDVAAVDPS-LLNYTAEE----------------------------

DM8C08G01580.1 NTSNGDQYDFNLVDGDVAAVDP--------------------------------------

DM8C08G01600.1 NTSNGDQYDFNLVDGDVAAVDPS-LLSISVATRLAQEDPAFASIKSLNYKQMLLLSLGTG

DM8C08G01600.1.2 NTSNGDQYDFNLVDGDVAAVDPS-LLSISVATRLAQEDPAFASIKSLNYKQMLLLSLGTG

DM8C08G01490.1 HTSNGDIYEFNLVDGGVATVGDPALLSLSVATKLAQVDPKFASIKSLDYKQMLLLSLGTG

DM8C08G01490.1.2 HTSNGDIYEFNLVDGGVATVGDPALLSLSVATKLAQVDPKFASIKSLDYKQMLLLSLGTG

DM8C08G01590.1 NTSNGDQYDFNLVDGDVAAV----------------------------------------

DM8C08G01670.1 NTSNGDKYEFNLVDGGVAAG----------------------------------------

DM8C08G01560.1 ------------------------------------------------------------

DM8C08G01530.1 NTSNGDQYDFNLVDGDVAAVDP--------------------------------------

DM8C08G01690.1 NTSNGDKYEFNLVDGGVAAGDPALLSVSVAMKHAENEDPAFASIKSLNYKKMLLLSLGTG

DM8C08G01690.1.2 NTSNGDKYEFNLVDGGVAAGDPALLSVSVAMKHAENEDPAFASIKSLNYKKMLLLSLGTG

X03932 NTSNGDKYEFNLVDGAVATVDDPALLSISVATKLAQVDPKFASIKSLNYKQMLLLSLGTG

DQ114417 HTSNGDRYEFNLVDGAVATVGDPALLSLSVATRLAQEDPAFSSIKSLDYKQMLLLSLGTG

DQ114419 HTSNGDKYEFNLVDGAVATVGDPALLSLSVATKLAQVDPKFASIKSLNYKQMLLLSLGTG

DQ114418 HTSDGDIYEFNLVDGAVATVGDPALLSLSVATRLAQEDPAFSSIKSLDYKQMLLLSLGTG

DQ114420 HTSNGDRYEFNLVDGGVATVGDPALLSLSVATKLAQVDPKFASIKSLDYKQMLLLSLGTG

DM8C08G01510.1 QRDLHKRIQHLLQLRKCIN-----------------------------------------

DM8C08G01620.1 TYRN--------------------------------------------------------

DM8C08G01650.1 --RIQHLLQLTQEAAKWGPLRWMLAIQQMTNAVSSYMTDYYISTVFQARHSQNNYLRVQE

DM8C08G01650.1.2 --RIQHLLQLTQEAAKWGPLRWMLAIQQMTNAVSSYMTDYYISTVFQARHSQNNYLRVQE

DM8C08G01480.1 TNSEFDKTHTAQETAKWGALQWMLVIQQMTEAASSYMTDYYLSTVFQDLHSQNNYLRVQE

DQ114415 TNSEFDKTHTAQETAKWGALQWMLVIQQMTEAASSYMTDYYLSTVFQDLHSQNNYLRVQE

NP_001275345 TNSEFDKTHTAQETAKWGALQWMLVIQQMTEAASSYMTDYYLSTVFQDLHSQNNYLRVQE

DQ114416 TTSEFDKTHTAEETAKWGALQWMLVIQQMTEAASSYMTDYYLSTVFQDLHSQNNYLRVQE

DM8C08G01660.1 ----------------------------------------------------------PE

DM8C08G01640.2 TNSEFAKNYTAEEAAKWGILQWMSPIWEMRSAASSYMNDYYLSTVFQALDSQNNYLRVQE

DM8C08G01500.1 ------------------------------------------------------------

DM8C08G01640.2.2 TNSEFAKNYTAEEAAKWGILQWMSPIWEMRSAASSYMNDYYLSTVFQALDSQNNYLRVQE

DM8C08G01630.1 TNSEFAKNYTAEEAAKWGILQWMSPLWEMRSAASSYMNDYYLSTVFQALDSQNNYLRVQE

DM8C08G01630.1.2 TNSEFAKNYTAEEAAKWGILQWMSPLWEMRSAASSYMNDYYLSTVFQALDSQNNYLRVQE

DQ114421 TNSEFAKNYTAEEAAKWGILQWMSPLWEMRSAASSYMNDYYLSTVFQALDSQNNYLRVQE

DM8C08G01540.2 TNSEFAKNYTAEEAAKWGILQWMSPIWEMRSAASSYMNDYYLSTVFQALDSQNNYLRVQE

DM8C08G01540.2.2 TNSEFAKNYTAEEAAKWGILQWMSPIWEMRSAASSYMNDYYLSTVFQALDSQNNYLRVQE

DM8C08G01550.1 -----------------------------------------------------------E

DM8C08G01570.1 ----------------------TIPLWEMRSAASSYMNDYYLSTVFQALDSQNNYLRVQE

DM8C08G01580.1 -----------------------------------------------------------E

DM8C08G01600.1 TNSEFAKNYTAEEAAKWGILQWMSPIWEMRSAASSYMNDYYLSTVFQALDSQNNYLRVQE

DM8C08G01600.1.2 TNSEFAKNYTAEEAAKWGILQWMSPIWEMRSAASSYMNDYYLSTVFQALDSQNNYLRVQE

DM8C08G01490.1 TNSEFDKTYTAEEAAKWGPLRWMLAIQQMTNAASSYMTDYYISTVFQARHSQNNYLRVQE

DM8C08G01490.1.2 TNSEFDKTYTAEEAAKWGPLRWMLAIQQMTNAASSYMTDYYISTVFQARHSQNNYLRVQE

DM8C08G01590.1 ------------------------------------------------------------

DM8C08G01670.1 ------------------------------------------------------------

DM8C08G01560.1 ------------------------------------------------------------

DM8C08G01530.1 -----------------------------------------------------------E

DM8C08G01690.1 TTSEFAKNYTAEEAAKWAIVQWILPLREMGNAASSYMNDYYLSTVFQALDSKNNYLRVQE

DM8C08G01690.1.2 TTSEFAKNYTAEEAAKWAIVQWILPLREMGNAASSYMNDYYLSTVFQALDSKNNYLRVQE

X03932 TTSEFDKTYTAEETAKWGTARWMLVIQKMTSAASSYMTDYYLSTAFQALDSQNNYLRVQE

DQ114417 TNSEFDKTYTAEEAAKWGPLRWLLAIQQMTNAASSYMTDYYLSTVFQARHSQNNYLRVQE

DQ114419 TNSEFDKTYTAEEAAKWGPLRWILAIQQMTNAASSYMTDYYLSTVFQARHSQNNYLRVQE

DQ114418 TNSEFDKTYTAEEAAKWGPLRWLLAIQQMTNAASSYMTDYYISTVFQAHHSQNNYLRVQE

DQ114420 TNSEFDKTYTAQETAKWGPLRWMLAIQQMTNAASSYMTDYYISTVFQARHSQNNYLRVQE

DM8C08G01510.1 ------------------------------------------------------------

DM8C08G01620.1 ------------------------------------------------------------

DM8C08G01650.1 NALTGTTTEMDDASEANMELLVQVGETLLKKPVSKDSP-ETYEEALKRFAKLLSDRKKLR

DM8C08G01650.1.2 NALTGTTTEMDDASEANMELLVQVGETLLKKPVSKDSP-ETYEEALKRFAKLLSDRKKLR

DM8C08G01480.1 NALTGTTTKADDASEANMELLVQVGENLLKKPVSKDNP-ETYEEALKRFAKLLSDRKKFR

DQ114415 NALTGTTTKADDASEANMELLVQVGENLLKKPVSKDNP-ETYEEALKRFAKLLSDRKKFR

NP_001275345 NALTGTTTKADDASEANMELLVQVGETLLKKPVSKDSP-ETYEEALKRFAKLLSDRKKLR

DQ114416 NALTGTTTKADDASEANMELLAQVGENLLKKPVSKDNP-ETYEEALKRFAKLLSDRKKLR

DM8C08G01660.1 NALTGSTTTWDNATLANMQLLKQVGENLLNKQVSKDNPDETYADALIG------------

DM8C08G01640.2 NTLTGTATKFDDASMANMILLVQVGENLLKKSVSEDNH-ETYEVALKRFAKLLSDRKKLR

DM8C08G01500.1 ---------------------VQVGENLLKKSVSEDNH-ETYEVALKRFAKLLSDRKKLR

DM8C08G01640.2.2 NTLTGTATKFDDASMANMILLVQVGENLLKKSVSEDNH-ETYEVALKRFAKLLSDRKKLR

DM8C08G01630.1 NALTGTATTFDDASVANMILLVQVGENLLKKSVSEDNH----------------------

DM8C08G01630.1.2 NALTGTATTFDDASVANMILLVQVGENLLKKSVSEDNH----------------------

DQ114421 NALTGTATTFDDASVANMILLVQVGENLLKKSVSEDNH-ETYEVALKRFAKLLSDRKKLR

DM8C08G01540.2 NALTGTATTFDDASVANMILLVQVGENLLKKSVSEDNH-ETYEVALKRFAKLLSDRKKLR

DM8C08G01540.2.2 NALTGTATTFDDASVANMILLVQVGENLLKKSVSEDNH-ETYEVALKRFAKLLSDRKKLR

DM8C08G01550.1 NALTGTATTFDDASVANMILLVQVGENLLKKSVSEDNH-ETYEVALKRFAKLLSDRKKLR

DM8C08G01570.1 NALTGTATTFDDASVANMILLVQVGENLLKKSVSEDNH-ETYEVALKRFAKLLSDRKKLR

DM8C08G01580.1 YTLTGTATKFDDASMANMILLVQVGENLLKKSVSEDNH-ETYEVALKRFAKLLSDRKKLR

DM8C08G01600.1 NALTGTTTTFDDASVANMILLVQVGENLLKKSVSEDNH-ETYEVALKRFAKLLSDRKKLR

DM8C08G01600.1.2 NALTGTTTTFDDASVANMILLVQVGENLLKKSVSEDNH-ETYEVALKRFAKLLSDRKKLR

DM8C08G01490.1 NALTGTTTEMDDASEANMDLLVQVGETLLKKPVSKDSP-ETYEEALKRFAKLLSDRKKLR

DM8C08G01490.1.2 NALTGTTTEMDDASEANMDLLVQVGETLLKKPVSKDSP-ETYEEALKRFAKLLSDRKKLR

DM8C08G01590.1 ------------------------DPQKRQLNG-------VFYNGCHLYGK---------

DM8C08G01670.1 ------------------------NPGVLKKSVSKDDP-ETYEEALKRFAKLLSDRKKLR

DM8C08G01560.1 ------------------------------------------------------------

DM8C08G01530.1 NALTGTATTFDDASVANMILLVQVGENLLKKSVSEDNH-ETYEVALKRFAKLLSDRKKLR

DM8C08G01690.1 NALTGTTTKMDDASVANMKLLEQVGKNLLKKNVSEDSH-ETYEVALKRFAKLLSDRKKLR

DM8C08G01690.1.2 NALTGTTTKMDDASVANMKLLEQVGKNLLKKNVSEDSH-ETYEVALKRFAKLLSDRKKLR

X03932 NALTGTTTELDDASEANMQLLVQVGEDLLKKSVSKDNP-ETYEEALKRFAKLLSDRKKLR

DQ114417 NALTGTTTEMDDASEANMELLVQVGETLLKKPVSKDSP-ETYEEALKRFAKLLSDRKKLR

DQ114419 NALTGTTTEMDDASEANMELLVQVGEKLLKKPVSKDSP-ETYEEALKRFAKLLSDRKKLR

DQ114418 NALTGTTTEMDDASEANMELLVQVGETLLKKPVSKDSP-ETYEEALKRFAKLLSDRKKLR

DQ114420 NALTGTTTEMDDASEANMELLVQVGETLLKKPVSKDSP-ETYEEALKRFAKLLSDRKKLR

DM8C08G01510.1 ----STATTFDDASVANMILLVQVGENLLKKSVSEDNH-ETYEVALKRFAKLLSDRKKLR

DM8C08G01620.1 ----------------------QFPKTIMKPMRLRGLQNCSLIRRNSEQTKRLF------

DM8C08G01650.1 ANKASY

DM8C08G01650.1.2 ANKASY

DM8C08G01480.1 ANKASY

DQ114415 ANKASY

NP_001275345 ANKASY

DQ114416 ANKASY

DM8C08G01660.1 ------

DM8C08G01640.2 ANKASF

DM8C08G01500.1 ANKASF

DM8C08G01640.2.2 ANKASF

DM8C08G01630.1 ------

DM8C08G01630.1.2 ------

DQ114421 ANKASF

DM8C08G01540.2 ANKASF

DM8C08G01540.2.2 ANKASF

DM8C08G01550.1 ANKASF

DM8C08G01570.1 ANKASF

DM8C08G01580.1 ANKASF

DM8C08G01600.1 ANKASF

DM8C08G01600.1.2 ANKASF

DM8C08G01490.1 ANKASY

DM8C08G01490.1.2 ANKASY

DM8C08G01590.1 ------

DM8C08G01670.1 ANKASY

DM8C08G01560.1 ------

DM8C08G01530.1 ANKASF

DM8C08G01690.1 ANKASY

DM8C08G01690.1.2 ANKASY

X03932 ANKASY

DQ114417 ANKASY

DQ114419 ANKASY

DQ114418 ANKASY

DQ114420 ANKASY

DM8C08G01510.1 ANKASF

DM8C08G01620.1 ------
